# Reviewers

**DOI:** 10.1111/jdi.12743

**Published:** 2017-11-03

The publication of invaluable papers in the Journal of Diabetes Investigation depends on the prompt, careful review of submitted manuscripts. We would like to thank the Editorial Board members, the International Editorial Board members, the Assistant Editorial Board members and the following experts for reviewing manuscripts from September 1, 2016 to August 31, 2017.

Norio Abiru

Harbeer Ahedi

Vasudha Ahuja

Yuichi Aita

Toru Aizawa

Kamel Ajlouni

Yasuhiro Akai

Paulo RS Amorim

Kai‐Nan An

Henning Andersen

Hitoshi Ando

Natalia Antonio

Naohiko Anzai

Atsushi Araki

Shin‐ichi Araki

Joao Ricardo Araujo

Hiroshi Arima

Noriko Asahara

Hiroo Asano

Yoshimasa Aso

Takuya Awata

Masayuki Baba

Tetsuya Babazono

Preethi Balan

Wei Bao

Helene Bihan

BW Bode

Ali Riza Cenk Celebi

Ali J Chakera

Katie Chan

Yi‐cheng Chang

Sanjay Chatterjee

Harn‐Shen Chen

Huang‐Chi Chen

Judy Chen

Liming Chen

Tseng‐Ji Chen

Yi‐Chuan Chen

Mei‐Ju Chi

Nino Cristiano Chilelli

Ken Chiu

Dong Hyeok Cho

Eun‐Hee Cho

Kai Chow Choi

Chih‐Hsun Chu

Yanhui Chu

Tsung‐Ju Chuang

Rachel Climie

Ayfer Colak

Miriam Giovanna Colombo

Hongyan Cui

Wenpeng Cui

Makoto Daimon

Takahisa Deguchi

Sagarika Ekanayake

Masanori Emoto

Liang Feng

Kazuya Fujihara

Shimpei Fujimoto

Tomomi Fujisawa

Yukihiro Fujita

Yoshio Fujitani

Maki Fukami

Michiaki Fukui

Tomoyasu Fukui

Shinya Furukawa

Hiroto Furuta

Jasvinder K Gambhir

Atsushi Goto

Siri Atma W Greeley

Honglei Guo

Xiaohui Guo

Kyoung Hwa Ha

Kazuyuki Hamaguchi

Yoshiyuki Hamamoto

Seung Jin Han

Toshiaki Hanafusa

Kazuo Hara

Shin‐ichi Harashima

Mitsuru Hashiramoto

Yuji Hataya

Toshio Hayashi

Yasuaki Hayashino

Jitsuo Higaki

Takumi Hirata

Takahisa Hirose

Inoue Hiroshi

Yushi Hirota

Yukio Horikawa

Masayuki Hosoi

Chang‐Hsun Hsieh

Hui‐Min Hsieh

Chia‐Luen Huang

Jee‐Fu Huang

Chii‐Min Hwu

Shunya Ikeda

Junta Imai

Reiko Inagi

Masahiko Inamori

Toyoshi Inoguchi

Manami Inoue

Mayumi Inoue

Yasushi Ishigaki

Hisamitsu Ishihara

Hideki Ishii

Hitoshi Ishii

Takahiro Ishikawa

Keisuke Ishizawa

Hesham A Issa

Masanori Iwanishi

Hideaki Jinnouchi

Shuji Joho

Keizo Kanasaki

Akio Kanazawa

Ippei Kanazawa

Hideaki Kaneto

Hae Yeon Kang

Le Kang

Yu‐Hsun Kao

Atsunori Kashiwagi

Sayaka Katagiri

Naoto Katakami

Koichi Kato

Shuichi Katoh

Tomoyuki Kawamura

Daiji Kawanami

Eiji Kawasaki

Yoshiaki Kido

Bu Kyung Kim

Chong Hwa Kim

Chul‐Hee Kim

Jae Hyeon Kim

Jaetaek Kim

Kyoung Soo Kim

Sang Soo Kim

Allen B King

Tadahiro Kitamura

Tetsuro Kobayashi

Motoyuki Kohjima

Mitsuhisa Komatsu

Masaya Koshizaka

Daisuke Koya

Nir Y Krakauer

Akira Kubota

Akio Kuroda

Takeshi Kurose

Minoru Kusama

Yoshiki Kusunoki

Tian Lan

Annunziata Lapolla

KP Law

Ting‐I Lee

CL Lee

De‐Chih Lee

I‐Te Lee

Mei‐Yueh Lee

Paul Lee

Yau‐Jiunn Lee

Yongho Lee

Jaechan Leem

Hung‐Yuan Li

X Li

Jun Liang

CH Lin

Kun‐Der Lin

Shi‐Dou Lin

Deshan Liu

Giieh‐Hua Lu

Xiaoping Luo

Norikazu Maeda

Shiro Maeda

Lucy Marzban

Hiroaki Masuzaki

Ikuro Matsuba

Masafumi Matsuda

Munehide Matsuhisa

Michihiro Matsumoto

Taka‐aki Matsuoka

Tomokazu Matsuura

Tomoya Mita

Hideaki Miyoshi

Tetsuya Mizoue

Hiroki Mizukami

Hasaan G Mohamed

Viswanathan Mohan

Lucia Monserrat Perez‐Navarro

Jun Sung Moon

Katsutaro Morino

Yuko Murase‐Mishiba

Takashi Murata

Yuki Murayama

Seiho Nagafuchi

Yoshio Nagai

Shoichiro Nagasaka

Hironori Nakagami

Tomoko Nakagami

Koichi Nakajima

Akinobu Nakamura

Keiko Nakamura

Nobuhisa Nakamura

Shuhei Nakanishi

Dmitry Namgaladze

Angela Napoli

Takuma Narita

José Navarro‐Partida

Masahiro Nishi

Nao Nishida

Wataru Nishie

Rimei Nishimura

Yoshihiko Nishio

Hitoshi Nishizawa

Hiroyuki Nodera

Claudia Cecilia Yamamoto Noguchi

Takashi Nomiyama

Atsushi Obata

Eiji Oda

Susumu Ogawa

Masahito Ogura

Mica Ohara‐Imaizumi

Ken Ohashi

Mitsuru Ohsugi

Yoichi Oikawa

Yosuke Okada

Tsuyoshi Okura

Altan Onat

Hideki Origasa

Tsuguhito Ota

Motoshi Ouchi

Nobuaki Ozaki

Cheol Whee Park

Yong‐Moon Park

Meda Pavkov

Milan Perovic

Panagiota Pietri

Yael D Reijmer

Sang Youl Rhee

Ohk Hyun Ryu

Yoshifumi Saisho

Kazuhiko Sakaguchi

Hiroshi Sakaue

Hiroshi Sakura

N Samir

Kazunori Sango

Tokio Sanke

Toshiyasu Sasaoka

Jo Satoh

Yusuke Seino

Naoto Seki

Fabrizio Sgolastra

Kenichi Shikata

Michio Shimabukuro

Akira Shimada

Hitoshi Shimano

Yuji Shimizu

Masashi Shimoda

Seiya Shimoda

Masakazu Shiota

Jun Shirakawa

Pierre Singer

Noriyuki Sonoda

Kevin Spencer

Paweł Stanirowski

Jasminka Stefulj

Jian‐bin Su

SC Su

Daisuke Sugiyama

Takashi Sugiyama

Sunghwan Suh

Naeti Suksomboon

Atushi Suzuki

Ryou Suzuki

Shigeru Suzuki

Kiyoshi Suzuma

Per‐Arne Svensson

Yuji Tajiri

Mitsuyoshi Takahara

Hirokazu Takahashi

Kazuma Takahashi

Toshinari Takamura

Shin Takasawa

Yoshiyu Takeda

Yoshifumi Tamura

Nagaaki Tanaka

Shoichiro Tanaka

Tetsuhiro Tanaka

Yukako Tatsumi

Ichiro Tatsuno

Shimosawa Tatsuo

Yasuo Terauchi

Jingyan Tian

Tatsuya Tominaga

Masao Toyoda

Satoru Tsujii

Daisuke Tsujino

Shin Tsunekawa

Kazuyo Tsushita

Yasuko Uchigata

Kohjiro Ueki

Hiroyuki Umegaki

Hiroyuki Unoki‐Kubota

Kazunori Utsunomiya

Takashi Uzu

Jay Vaidya

Claudia Vega Soto

Pragasam Viswanathan

Slavica Vuckovic

Jun Wada

Kaori Wakamatsu

Chih Yuan Wang

JS Wang

Changli Wei

Jong Chul Won

VW Wong

Yu Cho Woo

Chung‐Ze Wu

Jing Wu

Yen‐Wen Wu

Jianzhong Xiao

Zhangrong Xu

Toshihiko Yada

Hiroaki Yagyu

Satoru Yamada

Tomohide Yamada

Yuichiro Yamada

Sho‐ichi Yamagishi

Ritsuko Yamamoto‐Honda

Yasuhiko Yamamoto

Li Yan

Xilin Yang

Zuwei Yang

Hisafumi Yasuda

Hitoshi Yasuda

Abdullah Yeniova

Ester Chai Kheng Yeoh

Hirohide Yokokawa

Koichi Yokono

Masayasu Yoneda

Kun‐Ho Yoon

Hiroshi Yoshida

Narihito Yoshioka

Miao Yu

Xuefeng Yu

Hasniza Zaman Huri

Zhongheng Zhang

He‐qun Zou

